# Modeling and Optimization of MXene/PVC Membranes for Enhanced Water Treatment Performance

**DOI:** 10.3390/ma18153494

**Published:** 2025-07-25

**Authors:** Zainab E. Alhadithy, Ali A. Abbas Aljanabi, Adnan A. AbdulRazak, Qusay F. Alsalhy, Raluca Isopescu, Daniel Dinculescu, Cristiana Luminița Gîjiu

**Affiliations:** 1 Oil and Gas Refining Engineering Department, Al Hikma University College, Baghdad 10052, Iraq; zainabemadalhadithy@gmail.com; 2 Al Mussaib Technical College, Al-Furat Al-Awsat Technical University, Babylon P.O. Box 51006, Iraq; dr.ali.aljanabi@atu.edu.iq; 3 Membrane Technology Research Unit, Chemical Engineering Department, University of Technology, Al-Sinaa Street 52, Baghdad 10066, Iraq; adnan.a.alsalim@uotechnology.edu.iq (A.A.A.); qusay.f.abdulhameed@uotechnology.edu.iq (Q.F.A.); 4 Faculty of Chemical Engineering and Biotechnologies, National University of Science and Technology Politehnica Bucharest, 011061 Bucharest, Romania; raluca.isopescu@upb.ro (R.I.); daniel.dinculescu@upb.ro (D.D.)

**Keywords:** mixed matrix membrane (MMM), polyvinyl chloride (PVC), MXene, ultrafiltration, modeling, optimization

## Abstract

In this paper, MXene nanosheets were used as nano additives for the preparation of MXene-modified polyvinyl chloride (PVC) mixed max membranes (MMMs) for the rejection of lead (Pb^2+^) ions from wastewater. MXene nanosheets were introduced into the PVC matrix to enhance membrane performance, hydrophilicity, contact angle, porosity, and resistance to fouling. Modeling and optimization techniques were used to examine the effects of important operational and fabrication parameters, such as pH, contaminant concentration, nanoadditive (MXene) content, and operating pressure. Predictive models were developed using experimental data to assess the membranes’ performance in terms of flux and Pb^2+^ rejection. The ideal circumstances that struck a balance between long-term operating stability and high removal efficiency were found through multi-variable optimization. The optimized conditions for the best rejection of Pb^2+^ ions and the most stable permeability over time among the membranes that were manufactured were the initial metal ions concentration (2 mg/L), pH (7.89), pressure (2.99 bar), and MXene mass (0.3 g). The possibility of combining MXene nanoparticles with methodical optimization techniques to create efficient membranes for the removal of heavy metals in wastewater treatment applications is highlighted by this work.

## 1. Introduction

The increase in the discharge of heavy metals into water bodies due to industrial operations is a difficult environmental problem nowadays. Lead (Pb^2+^) ions are particularly harmful to aquatic ecosystems and human health because they are extremely toxic even at low concentrations [1,2]. Conventional techniques for removing Pb from wastewater such as chemical precipitation, ion exchange, and adsorption often have disadvantages such as insufficient removal, sludge production, and exorbitant operating expenditures [3].

Membrane technology has become a highly promising technique for purifying water because of its low energy consumption, high efficiency, and ease of operation [4]. Polyvinyl chloride (PVC), one of the several membrane materials, has drawn interest because of its high mechanical stability, affordability, and processing simplicity. To improve their effectiveness, PVC membranes must be modified due to their hydrophobic nature and moderate separation efficiency [5].

Although membranes offer certain advantages, they are prone to fouling and exhibit poor stability throughout the separation process [6]. A membrane’s utility can be enhanced by functionalizing and modifying it to boost performance attributes like selectivity and flux [7,8,9]. Incorporating nanomaterials such as MWCNT, TiO_2_, CNT, ZnO, MgO, and zeolite into the polymeric matrix was one of the significant advancements in enhancing the characteristic of polymer biodegradability [10,11,12,13,14].

Because of their distinct structure, large surface area, and adjustable surface chemistry, two-dimensional (2D) nanomaterials like MXenes have recently demonstrated exceptional promise in membrane research [15]. Recently, it has been shown that adding MXene to polymeric membranes significantly increases their hydrophilicity, antifouling capabilities, and contaminant rejection [16,17,18]. Notwithstanding its potential, MXene-based membranes’ manufacturing and operation parameters must be optimized in order to fully utilize their potential.

To improve the performance of MXene-based membranes, with the goals of achieving the best possible permeate flux, contaminant rejection, and operational stability over the long term, it is essential to find the best operating parameters [19,20].

Operating pressure, pH, feed solute concentration, and nanoadditive content are important variables that affect membrane efficiency [21]. For example, Ghadhban et al. [22] incorporated Hesperidin nanoparticles (HSP NPs) into (poly(lactic acid)/poly(butylene adipate-co-terephthalate)) for manufacturing mixed matrix membranes for oily wastewater treatment. To optimize the synthesis and operating parameters, they used an analysis of variance (ANOVA) and the response surface methodology (RSM). The results showed that, with 98.5316% oil removal and 121 L·m^–2^·h^–1^ flux, the PLA/PBAT/HSP MMMs had the best efficiency. The ideal parameters for the HSP MMMs to provide the best result were an HSP value of 0.03 weight percent, an oil concentration of 158.28 ppm, and a pressure of 3.5 bar [22].

The RSM, a helpful mathematical and statistical tool used to model and optimize the process, is influenced by a number of factors and interactions that affect the intended findings [23].

As a result, the response surface methodology (RSM) is used to analyze how input variables influence target responses and to determine the optimal conditions for achieving the desired performance [24,25,26].

While several recent studies [15,16,17,18,19,20] have investigated incorporating MXene into polymeric membranes for heavy metal removal, most have focused on direct blending or simple fabrication methods without systematic process optimization. In contrast, our work integrates MXene nanosheets into a PVC matrix and employs a comprehensive modeling and multi-variable and multi-objective optimization strategy using the response surface methodology (RSM). This approach enables predictive control over both fabrication and operational parameters such as pH, pressure, and contaminant concentration—resulting in an enhanced rejection efficiency and membrane stability. Thus, our method advances beyond previous MXene/PVC composites by providing a data-driven, optimized design framework specifically tailored for water treatment applications.

MXene nanosheets were added to PVC membranes in this study to enhance their ability to reject lead (Pb^2+^) ions from water. Four important variables, solution pH, Pb^2+^ content, MXene loading, and operating pressure, were used in a methodical modeling and optimization study. The effects of these parameters on water flux and Pb^2+^ rejection were assessed using multi-variable optimization and predictive modeling techniques in order to identify the ideal membrane performance conditions. This work adds to the expanding field of membranes strengthened by nanomaterials and provides information on how to adjust parameters for efficient heavy metal removal.

## 2. Materials and Methods

We used a 65 kg/mol molecular weight (MW) PVC polymer (≥98% purity) purchased from Georgia Gulf Co., Ltd., Atlanta, GA, USA. Sigma-Aldrich (St. Louis, MO, USA) supplied the N,N-dimethyl acetamide (DMAc) (99.8% purity), which was utilized as a solvent. Nanjing Aocheng Chemical Co., Ltd. (Nanjing, China) supplied ammonium hydrogen difluoride (NH_4_HF_2_) and titanium aluminium carbide (Ti_3_AlC_2_) with a 99.95% purity. Pb(NO_3_)_2_ was procured from Sigma-Aldrich (St. Louis, MO, USA).

## 3. Results

### 3.1. Membrane Manufacturing

The Ti_3_C_2_T_x_ MXene was synthesized from Ti_3_AlC_2_ MAX phase powder using a delamination method [27]. Briefly, 1 g of Ti_3_AlC_2_ was gradually added to 100 mL of 1 M ammonium hydrogen difluoride (NH_4_HF_2_) solution and stirred at 50 rpm and 60 °C for 12 h. The resulting slurry was centrifuged at 3500 rpm, ultrasonicated for 15 min to prevent aggregation, washed with ethanol and deionized water, vacuum filtered, and dried at 80 °C for 12 h.

**Figure 1:**
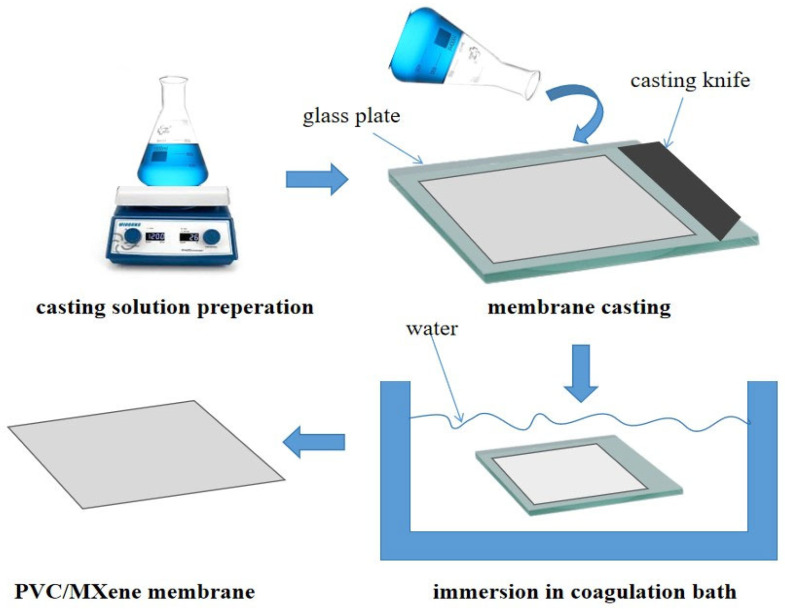
A schematic of the membrane fabrication process.

The non-induced phase inversion (NIPS) method was then used to create the neat and MXene-modified membranes as shown in Figure 1. To get rid of any remaining moisture, the PVC polymer was first dried. The membrane casting solution was made by dissolving a certain amount of PVC (14 weight percent) in 86 weight percent DMAC solvent while stirring continuously at 50 °C. After that, the mixture was then combined with various amounts of MXene, as shown in Table 1. To ensure total dissolution and homogeneity, the mixture was ultrasonically agitated for one hour and then stirred overnight. This technique is well known for dissolving agglomerates and promoting uniform nanosheet distribution in a polymer matrix. High-energy waves are introduced during sonication, which aids in the disintegration of particles and guarantees their uniform suspension in the PVC solution [28].

To get rid of the gas bubbles, the solution was then degassed. An automated casting procedure was used to cast the solution after it had been put on a glass substrate that had been cleaned and dehydrated (CX4 mtv messtechnik oHG, Erftstadt, Germany) in order to attain a 200 µm membrane thickness. The membrane was immediately immersed in a bath of water to solidify it. The fabricated membrane was removed at the completion of the NIPS technique, rinsed with deionized water to eliminate residual solvent, and subsequently stored in a water container for further analysis.

Preliminary optimization tests and data from the literature were combined to determine the polymer and solvent composition displayed in Table 1. By maintaining PVC’s adequate solubility in DMAc, this ratio produces a casting solution with a stable, homogeneous, and correct viscosity for the phase inversion procedure. An excessive concentration of PVC would make the solution more viscous, which would make membrane casting challenging and result in an uneven distribution of pores. On the other hand, a concentration that is too low might weaken the membrane’s mechanical properties. The selected composition achieves the best possible balance between permeability, solution processability, and mechanical characteristics [29,30].

The dispersion stability of MXene nanosheets within the PVC matrix was achieved by sonication and the use of DMF solvent to promote uniform mixing. No surfactants or surface modifications were employed. The stable and homogeneous distribution was confirmed by FE-SEM and EDX mapping (see Ref. [27]) and supported by the membrane’s stable performance during long-term filtration tests.

### 3.2. Membrane Performance

**Figure 2:**
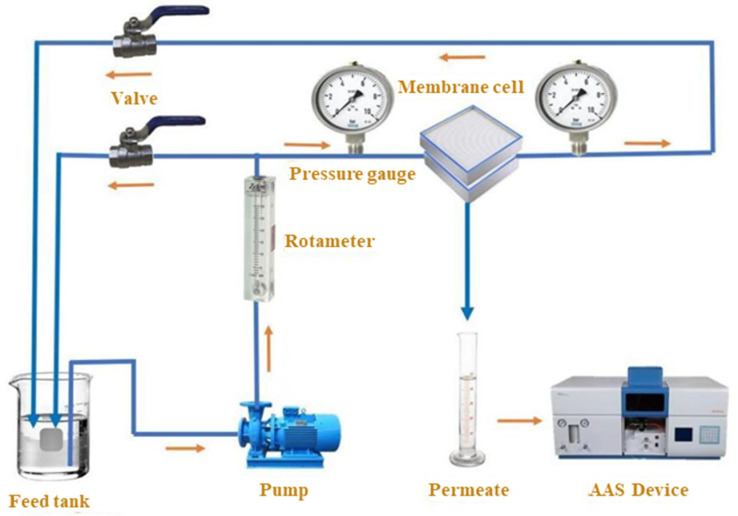
An ultrafiltration unit schematic image [27].

At 23 ± 1 °C, the membranes’ permeation flux and separation performance efficiency for Pb metal ions were assessed using a specially made cross-flow filtering apparatus (see Figure 2). The membrane’s surface area was 18 cm^2^. The water flux was calculated using the following equation [31]:

(1) $$J=\frac{V}{A\cdot t}$$

J stands for the water flux, which is expressed in L·m^–2^·h^–1^. A is the membrane surface area, and t is the duration for collecting the volume V.

Membrane rejection was evaluated with a Pb(NO_3_)_2_ aqueous solution. The amount of lead ions present can be tracked using atomic absorption spectroscopy (AAS). Equation (2) was utilized to determine the rejection of metal ions [32]:

(2) $$R\left(\%\right)=\left(1-\frac{C_{p}}{C_{f}}\right)\times 100$$

C_f_ is the concentration of the feed stream, whereas C_p_ is the concentration of the permeate stream. It is worth noting that the experimental characterization of the synthesized membranes, including morphological, structural, and electrical analyses (FE-SEM, EDX, XRD, and CA), is of great importance for further studies [33]. Our MXene-modified membrane was previously fully characterized and published in our earlier work [27]. The present study builds upon these results to develop and optimize a modeling framework.

### 3.3. Optimization and Modeling

In order to gain a deeper understanding of how the operational parameters and attributes affect the performance of the blend membranes, we undertook optimization and mathematical modeling. This was essential for understanding the impact of operational parameters on membrane performance, considering the membrane properties that were available. This helped to enhance our comprehension of the overall process.

RSM is a statistics and mathematics method for designing experiments. The goal is to improve the process which is impacted by multiple independent factors [34]. Another benefit of using an RSM design is the reduced need for trials compared to a full experimental design at the same level. Moreover, the primary objective of the response surface methodology is to determine the most favorable operating circumstances for the system or to pinpoint the specific range that satisfies the operating criteria. Regression analysis has been used to assess how independent factors affect the dependent variables. This technique enables a more rigorous estimation of the optimal conditions of process operating variables by analyzing the factors’ relative relevance [35].

In order to assess their effects on the feed and rejection percentage of the membranes, the current study focused on optimizing the operational parameters, including the concentration of lead (2 to 10 ppm), the pressure across the membrane (1 to 3 bars), the pH (4 to 10), and using a range of 0–0.4 g for the MXene nanosheet.

As experimentally proven [27], 0.5 g of MXene led to agglomeration and decreased performance; 0.4 g remained within the stable range. Therefore, this concentration was included in the modeling to capture the membrane behavior near the upper dispersion limit.

Furthermore, this work analyzed the impact of functional parameters on the results. The ANOVA (analysis of variance) is a statistical technique used to evaluate the efficacy of constructed models using statistical data and evaluation tests [36]. Table 2 presents the empirical data and symbols used to represent variables. A series of studies were performed utilizing ultra-filtration membrane technology. Each test involved altering a single element to determine the necessary operational parameters while maintaining all other factors at constant.

The objective of the optimization technique (RSM) is to analyze the influence of independent factors, sometimes called predictors, on the flux and rejection percentage in the separation process, which are the responses being studied. Table 2 presents the range of parameter variation within the experimental study. The details of the experiments conducted as a part of this investigation are shown in Table 3.

## 4. Discussion

### 4.1. Evaluation of MMMs Filtration Cross-Flow

The pure water flow (PWF) of each mixed matrix membrane (MMM) was higher than that of the pristine PVC membrane (N0). Even at relatively modest loading concentrations, the flow parameters were significantly affected by the addition of MXene nanosheets. The experimental findings demonstrated that the addition of 0.1 g MXene (N1) raised the water flux from 128 L·m^–2^·h^–1^ for the unmodified membrane (N0) to 170 L·m^–2^·h^–1^. The PWF increased with additional MXene incorporation, peaking at 201 L·m^–2^·h^–1^ for membrane N2, which had 0.4 g MXene.

The improvements in surface hydrophilicity, pore size distribution, and porosity seen in the MXene-modified membranes were in good correlation with the increase in PWF, as was previously mentioned in this work. Nevertheless, the PWF decreased to roughly 118.5 L·m^–2^·h^–1^ when the MXene content was raised to 0.5 g (N3). The potential aggregation of excess MXene inside the membrane matrix was thought to be the cause of this reduction, since it probably resulted in pore obstruction and increased resistance to water transport [37]. The stability of MXene nanosheets within the membrane matrix was confirmed by leaching tests reported in our earlier work [27], which showed that MXene release stabilized at approximately 5.2% after three weeks of soaking, indicating minimal nanoparticle leaching. The antifouling performance of the membranes was assessed through flow recovery ratio (FRR) measurements and multi-cycle filtration tests. MXene-modified membranes exhibited significantly higher FRR values and superior flux stability over three cycles compared to bare PVC membranes, demonstrating enhanced fouling resistance and improved operational stability [38].

### 4.2. ANOVA Results

An analysis of variance (ANOVA) yields *p*-values for each design-layout predictor variable, which are based on the transmembrane pressure values, MXene content, pH, and Pb concentration. These results are shown in Table 4 and Table 5 for flux (L·m^–2^·h^–1^) and rejection (%), respectively.

In order to develop a mathematical model that could forecast the process outcome, the results were examined. Equations (3) and (4) for membranes, in terms of the actual variables, respectively, represent the regression model equations for flow and rejection (%).

(3) $$\begin{array}{ll} \mathrm{Flux}\;\left(\frac{L}{m^{2}\cdot h}\right) & =6.8046+1.8648\cdot A+16.1872\cdot B+7.5734\cdot C-377.9060\cdot D-0.0219\cdot A\cdot B\\ & -\;0.0128\cdot A\cdot C+0.0971\cdot A\cdot D+0.9517\cdot B\cdot C+74.4447\cdot B\cdot D\\ & +\;2.3205\cdot C\cdot D-0.1618\cdot A^{2}-0.4534\cdot B^{2}-0.5670\cdot C^{2}+1450.7904\cdot D^{2} \end{array}$$

(4) $$\begin{array}{ll} \mathrm{Rejection}\;\left(\%\right) & =41.6219+1.9621\cdot A+2.6609\cdot B+9.1457\cdot C+141.6780\cdot D-0.0338\cdot A\cdot B\\ & -\;0.0263\cdot A\cdot C-4.6531\cdot A\cdot D-0.0700\cdot B\cdot C-0.1375\cdot B\cdot D\\ & -\;0.2458\cdot C\cdot D-0.2418\cdot A^{2}-0.8691\cdot B^{2}-0.6799\cdot C^{2}-172.9781\cdot D^{2} \end{array}$$

where the flux is denoted by F (L·m^–2^·h^–1^), A represents the concentration of Pb in the feed (ppm), B represents the pressure in bars, C represents the level of pH, D is the quantity of MXene in (g), and R (%) the rejection percentage.

The determination coefficient R^2^ was found to be 99.68% for the flux and 98.45% for the rejection. Additionally, the modified R^2^ values are 97% and 99.38%, respectively, which strongly agree with the R^2^ values and are appropriately high. The information shows that the model obtained for the flow and rejection percentage is statistically sound and capable of correctly forecasting UF membrane performance.

According to the ANOVA results, some variable coefficients have extremely high *p*-values, meaning that these terms can be neglected without losing the capacity of model prediction. Considering these aspects, the two models were simplified, renouncing to highly insignificant contributions. The reduced new regression model for flux is given by the following expression:

(5) $$\begin{array}{ll} \mathrm{Flux}\;\left(\frac{L}{m^{2}\cdot h}\right) & =8.1066+1.8513\cdot A+14.2421\cdot B+7.7050\cdot C-375.9838\cdot D+0.9517\cdot B\cdot C\\ & +\;74.4447\cdot B\cdot D+2.3205\cdot C\cdot D-0.1701\cdot A^{2}-0.5819\cdot C_{3}^{2}+1447.4415\cdot D^{2} \end{array}$$

The determination coefficient is at a high level (R^2^ = 99.68%) and so is the adjusted R square (Adj R^2^ = 99.50 %), showing that the reduced regression model can well reflect the variation of flux with operating parameters.

The reduced model for rejection is as follows:

(6) $$\begin{array}{ll} \mathrm{Rejection}\;\left(\%\right) & =43.4060+1.8946\cdot A+1.9409\cdot B+8.9565\cdot C+139.6822\cdot D-0.0263\cdot A\cdot C\\ & -\;4.6531\cdot A\cdot D-0.2418\cdot A^{2}-0.8691\cdot B^{2}-0.6799\cdot C^{2}-172.9781\cdot D^{2} \end{array}$$

**Figure 3:**
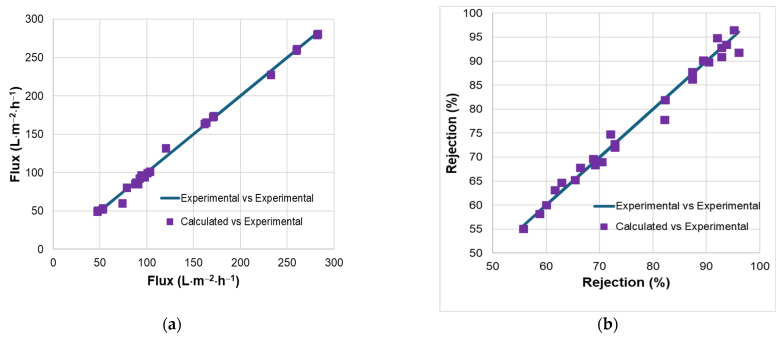
Parity plots for experimental and calculated values of flux (**a**) and rejection (**b**).

With the R^2^ = 98.42% and adjusted R^2^ = 97.59%, both are very close to the complete regression model. The capacities of the two reduced regression models to capture the variation of the flux and rejection are also proved by the parity plots (Figure 3).

### 4.3. Process Optimization

As the main objective of the present study is to obtain optimum operating conditions that ensure simultaneously maximum flux and maximum rejection, a multi-objective optimization problem must be defined.

For such an attempt, two main strategies were used in this work:

(i) Lump the two criteria by defining a weighted sum of the two objectives (relation 7) and the maximization of this lumped function.

(7) $$\;F(x)=\omega_{1}\cdot f_{1}(x)+\omega_{2}\cdot f_{2}(x)$$

In this case, the solution depends on the wights ω_i_ assumed for each basic criterion.

(ii) The Pareto-based approach method aims to find a set of Pareto optimal solutions rather than a single optimal solution.

Firstly, the two reduced regression models were used in the formulation of the compound objective function (Equation (7)), where x stands for the vector of operating variables x = [A, B, C, D], while f_1_(x) and f_2_(x) are the flux and rejection, respectively, calculated with the reduced regression models (Equations (5) and (6)).

The maximization was carried out in the frame of the Matlab 2024 environment using the built-in function *fmincon*, designed for the minimization of objective functions with restrictions, using the interior point algorithm. As *fmincon* performs the minimization of objective functions, the lumped function F(x) defined by Equation (7), with the changed sign considered in the procedure, as a maximum of the two criteria is searched. The upper and lower bonds for the variables were defined according to the experimental plan:

The optimum solution obtained was A = 2.03 ppm, B = 3 bar, C = 8.17, and D = 0.4 g, for which the flux was 279.81 L·m^–2^·h^–1^ and rejection was 96.86%.

**Figure 4:**
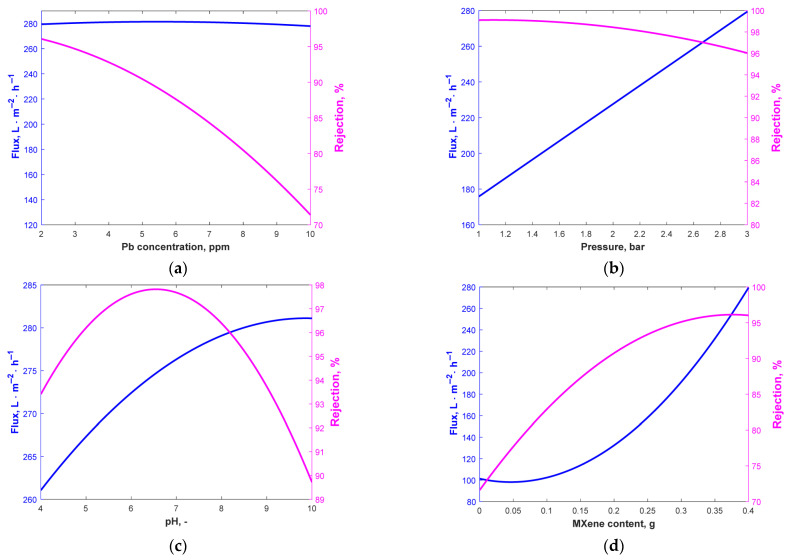
Flux and rejection variation with Pb concentration for (**a**) B = 3 bar, C = 8.17, D = 0.4 g; (**b**) A = 2.03 ppm, C = 8.17, D = 0.4 g; (**c**) A = 2.03 ppm, B = 3 bar, D = 0.4 g; (**d**) A = 2.03 ppm, B = 3 bar, C = 8.17.

The visualization of each variable effect upon the two main measured outcomes (the flux and rejection coefficient) are represented in Figure 4, where the evolution of the two outcomes is represented for each factor, considering the other three operating parameters at the values identified in the optimization step.

As can be noticed, the flux is less influenced by Pb concentration, while the rejection coefficient is clearly favored by low Pb concentration values. The pressure influences both process outcomes, but in opposite ways. As the rejection coefficient is less influenced by the pressure variation (for the whole investigated range, the rejection is over 96%), the optimum value previously determined recommended 3-bar intermembrane pressure, as the flux increases significantly with increased pressure. The pH clearly influences the flux and rejection. The flux would have a maximum value at pH > 9 while the rejection would be the highest at a pH between 6 and 7. Because it has an impact on the dissociation and ionization of Pb particles, solution pH is a crucial factor influencing the removal process. Altering the pH of the fluid can modify the membrane’s surface charge, resulting in several outcomes. Metal rejection is influenced by pH, as higher pH levels enhance the interaction between metal ions and polymeric ligands.

The complex’s lifetime is limited to low pH because of the poor affinities that attach to the ions of metal due to its lack of electric-positive charge. The stability and affinity of binds with metal complexes increase with pH. However, metal hydroxide precipitation is more likely to occur at neutral or alkaline pH levels. The findings of Alpatova et al. [39] and Arthanareeswaran et al. [40] on the removal of heavy metals using complexation ultrafiltration were consistent with the present data. They stated that at pH values greater than 6, the precipitation of metal hydroxide causes the heavy metal ions to be entirely maintained on the membrane. The higher tendency of Pb^2+^ precipitation in neutral- or low-alkaline environments may justify the higher coefficient of rejection and a lower value of the flux that is caused by the blocking of the membrane pores in these working conditions. As can be noticed from Figure 4d, the addition of MXene is favorable for the flux and rejection coefficients.

**Figure 5:**
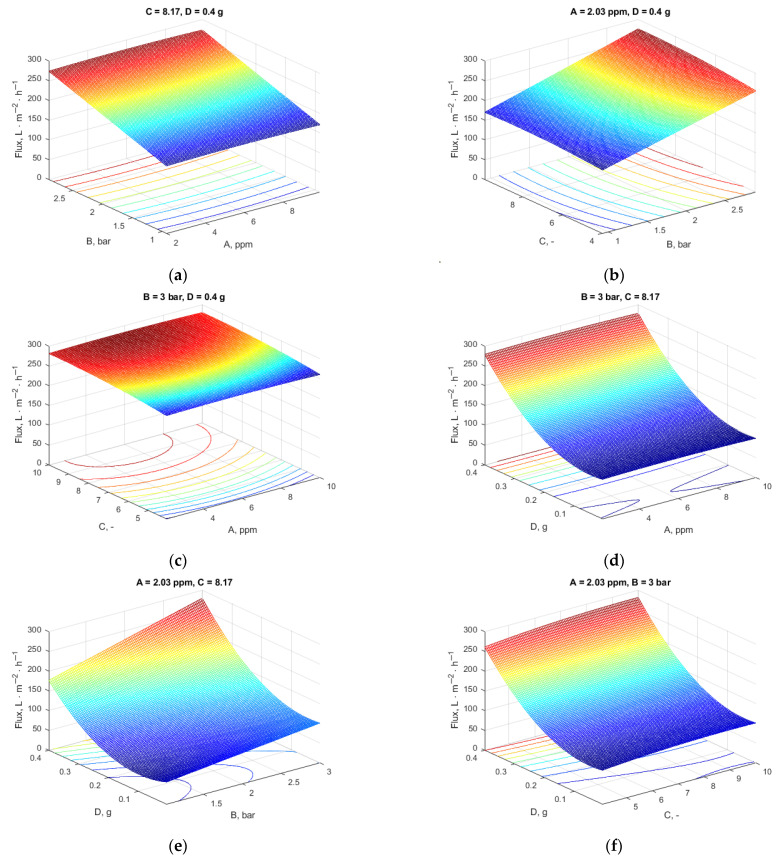
Influence of variables on the flux (two variables were kept at the values resulting from the optimization): (**a**) constant pH and additive content; (**b**) constant Pb concentration and additive content; (**c**) constant pressure and additive loading; (**d**) constant pressure and pH; (**e**) constant Pb concentration and pH; (**f**) constant Pb concentration and pressure.

**Figure 6:**
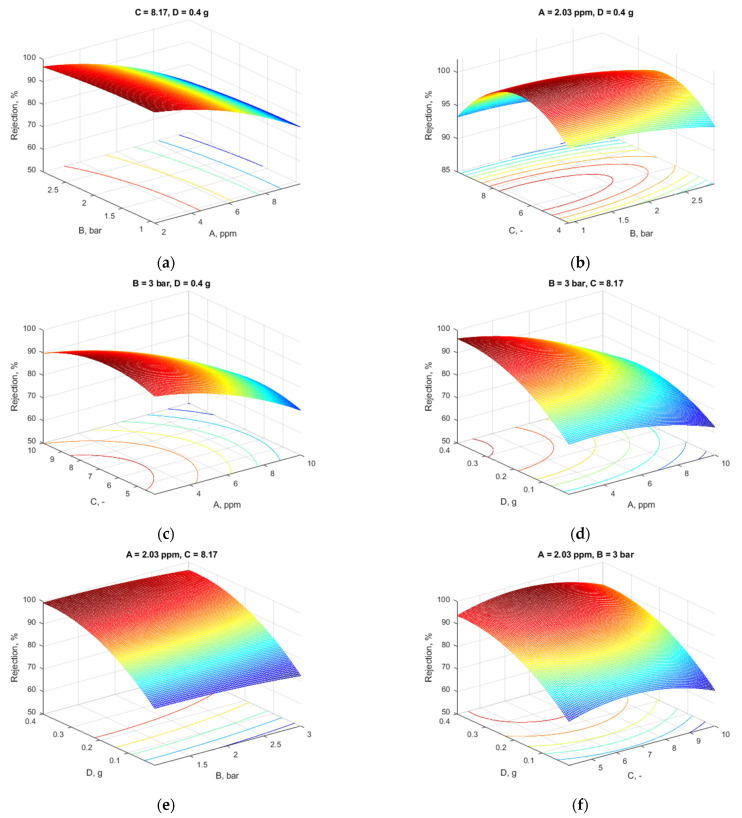
Influence of variables on the rejection (two variables were kept at the values resulting from the optimization): (**a**) constant pH and additive content; (**b**) constant Pb concentration and additive content; (**c**) constant pressure and additive loading; (**d**) constant pressure and pH; (**e**) constant Pb concentration and pH; (**f**) constant Pb concentration and pressure.

To visualize the multiple effects of variables upon the flux and rejection, 3D graphical representations of the response surfaces were obtained, maintaining two of the four variables at constant values according to the optimum solution. Figure 5 represents the flux variation and Figure 6 reflects the influence of process parameters upon the rejection.

### 4.4. Response Surface Analysis

From Figure 5a, for pressure and Pb concentration, it is clear that the Pb concentration had the lowest effect on the flux as the plane was parallel because of the low-concentration differences between them.

Figure 5b indicates that the flux increased with increasing pressure and decreasing pH, and, as previously explained, the MXene content and Pb concentration were constant. The contour plots’ inherent nature shows no interaction between these factors (parallel straight lines).

Figure 5c depicts the Pb concentration and the solution’s pH influence. The plot indicates that the flow was positively impacted by the pH. As the pH of the solution rose, so did the flow.

Figure 5d presents the effect of the two factors: Pb concentration and MXene nanosheet quantity when pH and pressure were held at 8.03 bar and 3 bar, respectively. As shown in this figure, the increase in MXene loading led to a rise in flux up to 290 L·m^–2^·h^–1^, while the variance in Pb concentration had a less significant influence on the flux.

The sort of interaction between MXene quantity and pressure is seen in Figure 5e. The flow increased more than the pressure when the additive content was raised to 0.4 g. The effects of increasing the MXene content, the membrane’s porosity, average pore size, and hydrophilicity, as well as raising the transmembrane pressure, all work together to increase the permeate flux by raising the membrane surface’s shear stress.

At a pressure of 3 bar and a constant Pb concentration of 2.09, Figure 5f shows that increased flow was produced by increasing the MXene quantity and pH.

Figure 6a shows how pressure and Pb concentration affect the rejection. As the quantity of metal ions rose, the metal rejection decreased gradually, a maximum value of the metal rejection being noticed at 2 ppm Pb (as evidenced also in Figure 6c,d). This is in accordance with the Irving–Williams series, which ranks the divalent metal ions in their tendency to complexity [41]. Elevating the pressure resulted in a minor decrease in membrane rejection. Consequently, an increase in pressure may induce deformation in polymer chains and enlarge pore size due to pore obstruction or the production of a resistive layer on the membrane’s surface. The rejection rate marginally diminished at elevated pressures. An increase in Pb concentration results in a drop in the rejection value.

In Figure 6b, the effect of pH and pressure is depicted at a constant Pb concentration of 2.09 ppm and an additive content of 0.4 g. It is observed that increasing pressure resulted in less rejection of metal ions. The pH had a less significant influence on rejection.

Figure 6c presents the influence of the two factors, pH and Pb concentrations, when the pressure and MXene loading were held at 3 and 0.4 g, respectively. As can be noticed in the figure, the retention coefficient was favored by small values of Pb concentrations, as the rise of Pb concentration up to 10 ppm led to a significant decrease in the retention, while the pH had a less significant influence on rejection. A maximum of retention coefficient was noticed for a pH between 6 and 8, which is also shown in Figure 6b. The values recorded registered for both flux and rejection were obtained at a pH above 7.

Figure 6d depicts the Pb concentration, and the concentration of the additive influenced the flux at a constant pH and pressure. From the plot, the rejection of Pb was positively impacted by the additive concentration. As the membranes’ additive content rose, so did the rejection. This was due to the fact that a high additive concentration (0.4 g) resulted in a high rejection for heavy metal ions, which permitted an interaction between the Pb ions and the accessible function group on the membrane surface.

The response surface plot in Figure 6e shows how MXene content and pressure affected rejection at a constant Pb concentration of 2.09 ppm and pH 8.09. While increasing the pressure reduced the rejection, increasing the MXene loading to 0.4 g increased rejection, even at a high pressure.

In Figure 6f is depicted the effect of two factors, MXene content and pH, at a constant Pb concentration of 2.09 ppm and a pressure of 3 bar. As can be observed from the figure, the increase in MXene content led to an increase in rejection, while the variance of MXene content had a very high, significant effect on rejection.

### 4.5. Pareto Front Building as Multi-Objective Optimization Technique

In multi-objective optimization, a non-dominated solution (also known as a Pareto optimal solution) refers to a solution that is not dominated by any other solution in the solution set. In simple terms, it means that no other solution exists that is better in all objective functions. The Pareto front is practically a set of equivalent optimal solutions which allows for the choice of some operating parameter combination that can realize the trade-off between maximum flux and the maximum retention coefficient.

Using Matlab2024′s genetic algorithm (GA), the Pareto front was produced. The polynomial models derived from the experimental design and regression analysis (Equations (5) and (6)) expressed the goal functions taken into consideration.

**Figure 7:**
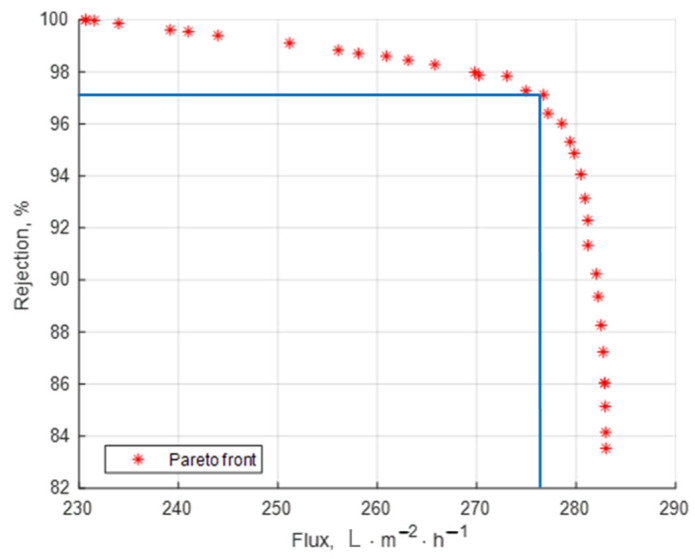
Pareto front with optimal solutions.

The outcomes are displayed in Figure 7.

Table 6 presets some possible optimal solutions selected from the Pareto front.

If the rejection must be very high, for instance, over 98%, then another set of operating parameters should be chosen even if some reduction in the flux may occur (see the solution in line 1 of Table 6).

If the flux is the highest priority, the selected solution on the front should correspond to a high value of the flux, accepting a reasonable reduction in the rejection coefficient (solution in line 2 of Table 6).

If the two objectives are of similar importance the user may choose the operating point marked in Figure 7. In the present study, this operating point was very close to the optimal solution already obtained by using the lumped objective function (Equation 7) where the weights of the two criteria were equal (solution in line 3 of Table 6).

## 5. Conclusions

This work examined the successful fabrication and systematic evaluation of MXene-modified polyvinyl chloride (PVC) mixed matrix membranes (MMMs) for the rejection of lead (Pb^2+^) ions from aqueous solutions. To improve membrane performance, important operating and fabrication parameters were modeled and optimized, such as pH, feed contaminant concentration, nanoadditive (MXene) content, and applied pressure.

Pure water flow, porosity, and membrane hydrophilicity were all markedly enhanced by the addition of MXene. When compared to the neat PVC membrane, the membrane with 0.4 g MXene (N2) performed the best among the manufactured membranes, achieving a significant improvement in water flux and Pb^2+^ rejection. Higher MXene loading over the ideal content, however, caused agglomeration and reduced membrane permeability.

Finding the optimum operating conditions for maximum rejection efficiency and long-term stability was made possible by the adoption of a methodical modeling and optimization technique, which offered insightful information about the intricate relationships between operational variables. Also, the multi-objective approach provides a set of trade-off choices for operating conditions to achieve simultaneous maximum rejection–maximum permeate flux.

These findings underscore the significance of optimization strategies in developing membrane processes and indicate that MXene-based mixed matrix membranes (MMMs) could be a practical choice for removing heavy metals in wastewater treatment.

This study focused on synthetic Pb^2+^ solutions to facilitate controlled modeling and parameter optimization. Future research will evaluate the effects of coexisting ions and complex wastewater compositions on membrane performance and rejection selectivity.

## Figures and Tables

**Table 1:** The membrane casting solution’s PVC, DMAc, and MXene contents.

Membrane Code Weight	N0	N1	N2	N3
PVC %	14	14	14	14
DMAc	86	86	86	86
MXene (g)	0	0.1	0.4	0.5

**Table 2:** The different levels of experimental variables.

Parameters	Code	Unit	Low Level	High Level
Pb concentration	A	ppm	2	10
Pressure	B	bar	1	3
pH	C	−	4	10
MXene content	D	g	0	0.4

**Table 3:** Experimental design data in relation to flux and rejection.

Run Number	A: Pb Concentration (ppm)	B: Pressure (bar)	C: pH (−)	D: MXene Content (g)	Flux (L·m^–2^·h^–1^)	Rejection (%)
1	6	2	10	0.2	94.02	82.3
2	10	1	4	0	47.25	61.6
3	2	3	10	0	103.67	65.4
4	10	3	10	0.4	282	62.9
5	10	1	4	0.4	161.72	72.8
6	2	1	10	0	53.05	69.2
7	6	2	7	0.2	94.54	89.4
8	2	3	4	0.4	260.05	93.76
9	6	2	7	0.2	94.54	89.4
10	6	2	7	0.2	94.54	89.4
11	10	2	7	0.2	92.33	82.2
12	6	2	4	0.2	91	87.4
13	6	2	7	0.2	94.54	89.4
14	6	2	7	0.2	94.54	89.4
15	2	1	4	0.4	163.33	95.2
16	10	3	4	0	87.407	60
17	2	3	10	0.4	282.83	90.4
18	10	1	10	0	52.55	58.8
19	6	3	7	0.2	120.47	87.4
20	6	2	7	0	78.67	72
21	6	2	7	0.2	94.54	89.4
22	10	3	4	0.4	260	68.8
23	2	2	7	0.2	97.72	92
24	6	2	7	0.4	232.62	96.1
25	2	1	10	0.4	171.47	92.8
26	2	3	4	0	88.38	70.4
27	10	1	10	0.4	170.42	66.4
28	6	1	7	0.2	73.85	92.8
29	10	3	10	0	100.66	55.7
30	2	1	4	0	47.55	72.8

**Table 4:** ANOVA study for the flux.

Source	Sum of Squares	DF	Mean Square	*p*-Value	F-Value
Model	1.44 × 10^5^	14	10,335.3	<0.0001	331.3
A-Con.	10.4	1	10.4	0.571	0.3
B-Press	23,060.7	1	23,060.7	<0.0001	739.4
C-pH	600.6	1	600.6	0.0005	19.2
D-Additives	97,571.9	1	97,571.9	<0.0001	3128.4
AB	0.1	1	0.1	0.9508	3.94 × 10^−3^
AC	0.3	1	0.3	0.9139	0.01
AD	0.09	1	0.09	0.9564	3.09 × 10^−3^
BC	130.4	1	130.4	0.0588	4.1
BD	3546.8	1	3546.8	<0.0001	113.7
CD	31.0	1	31.0	0.334	0.9
A2	17.3	1	17.36	0.467	0.56
B2	0.5	1	0.53	0.897	0.01
C2	67.4	1	67.4	0.162	2.1
D2	8725.3	1	8725.3	<0.0001	279.7
Residual	467.8	15			
Lack of Fit	467.83	10			
Pure Error		5			
Cor Total	1.45 × 10^5^	29			
Model	Std. Dev.		R-Squared	Pred. R-Squared	Adj R-Squared
Summary	5.58		0.9968	0.9862	0.9938

**Table 5:** ANOVA study for the Pb rejection.

Source	Sum of Squares	DF	Mean Square	*p*-Value	F-Value
Model	4797.6	14	342.6	<0.0001	68
A-Con.	1296.4	1	1296.4	<0.0001	257.2
B-Press	42.4	1	42.4	0.0109	8.4
C-pH	83.8	1	83.8	0.0010	16.6
D-Additives	1304.9	1	1304.9	<0.0001	258.9
AB	0.2	1	0.2	0.813	0.05
AC	1.5	1	1.5	0.582	0.3
AD	221.7	1	221.7	<0.0001	44
BC	0.7	1	0.7	0.713	0.1
BD	0.01	1	0.01	0.961	2.40 × 10^−3^
CD	0.3	1	0.3	0.796	0.06
A2	38.7	1	38.7	0.014	7.7
B2	1.9	1	1.9	0.542	0.3
C2	97	1	97	0.0005	19.2
D2	124	1	124	0.0002	24.6
Residual	75.5	15	5		
Lack of Fit	75.5	10	7.5		
Pure Error		5			
Cor Total	4873.2	29			
Model	Std. Dev.		R-Squared	Pred. R-Squared	Adj R-Squared
Summary	2.24		0.9845	0.9303	0.9700

**Table 6:** Selected optimal solutions.

A, ppm	B, bar	pH	D, g	Flux, L·m^–2^·h^–1^	Rejection, %	Comments
2.0442	2.92	6.74	0.3995	270.7711	98.05	A decrease of less than 3% in flux
3.3719	2.99	9.10	0.3998	281.6565	91.38	Gain in flux, 5% loss in rejection
2.0174	2.99	7.89	0.3998	278.4888	96.61	Optimal point for criteria equally important
2.0152	2.49	6.61	0.3991	248.2754	99.29	Very high rejection but unacceptably low flux value (in agreement with Figure 4b,c)

## Data Availability

The original contributions presented in this study are included in the article. Further inquiries can be directed to the corresponding author.
